# Supplementary material for: Production of β-Glucans from Rhizopus oryzae M10A1 by Optimizing Culture Conditions Using Liquid Potato Starch Waste
Source: Polymers (Basel). 2025 May 7;17(9):1283. doi: 10.3390/polym17091283 (PMC12074454; doi:10.3390/polym17091283)
Supplement: Supplementary file 1 [file polymers-17-01283-s001.zip › polymers-3492415-supplementary.pdf]

Table S1. Characterization of culture media used in the optimization of β-glucans from *Rhizopus oryzae* M10A1.

Run	Total solids (g/100mL)		Suspension solids (g/100mL)		Soluble solids (g/100mL)		Ash (g/100mL)	
	Before cultivation	After cultivation	Before cultivation	After cultivation	Before cultivation	After cultivation	Before cultivation	After cultivation
1	2.84 ± 0.08a	1.20 ± 0.08b	2.40 ± 0.02a	0.82 ± 0.09b	0.44 ± 0.06a	0.38 ± 0.06b	0.35 ± 0.00a	0.30 ± 0.02b
2	3.28 ± 0.07a	1.25 ± 0.02b	1.56 ± 0.10a	0.89 ± 0.18b	1.72 ± 0.03a	0.36 ± 0.03b	0.34 ± 0.04a	0.32 ± 0.02b
3	3.27 ± 0.07a	1.38 ± 0.01b	2.76 ± 0.13a	1.40 ± 0.04b	0.51 ± 0.05a	0.87 ± 0.01b	0.30 ± 0.02a	0.24 ± 0.00b
4	3.48 ± 0.05a	1.65 ± 0.03b	2.95 ± 0.15a	1.50 ± 0.08b	0.53 ± 0.07a	0.15 ± 0.10b	0.31 ± 0.04a	0.26 ± 0.00b
5	2.71 ± 0.31a	1.35 ± 0.01b	2.53 ± 0.11a	1.28 ± 0.06b	0.18 ± 0.02a	0.07 ± 0.02b	0.30 ± 0.03a	0.32 ± 0.01b
6	3.45 ± 0.05a	1.68 ± 0.11b	2.38 ± 0.11a	1.60 ± 0.02b	1.07 ± 0.09a	0.08 ± 0.03b	0.35 ± 0.02a	0.35 ± 0.00b
7	3.04 ± 0.05a	1.37 ± 0.04b	2.67 ± 0.24a	1.19 ± 0.01b	0.37 ± 0.04a	0.18 ± 0.01b	0.23 ± 0.01a	0.13 ± 0.02b
8	3.27 ± 0.01a	1.84 ± 0.08b	2.90 ± 0.03a	1.71 ± 0.01b	0.37 ± 0.02a	0.13 ± 0.05b	0.35 ± 0.02a	0.30 ± 0.03b
9	3.33 ± 0.07a	1.48 ± 0.07b	2.78 ± 0.05a	1.26 ± 0.08b	0.55 ± 0.01a	0.22 ± 0.05b	0.36 ± 0.01a	0.33 ± 0.04b
10	3.34 ± 0.05a	1.46 ± 0.08b	2.77 ± 0.03a	123 ± 0.02b	0.57 ± 0.02a	0.23 ± 0.01b	0.38 ± 0.02a	0.32 ± 0.02b
11	3.32 ± 0.08a	1.47 ± 0.09b	2.76 ± 0.08a	1.20 ± 0.00b	0.56 ± 0.10a	0.27 ± 0.00b	0.37 ± 0.03a	0.31 ± 0.00b
12	3.31 ± 0.06a	1.46 ± 0.03b	2.75 ± 0.04a	1.25 ± 0.08b	0.56 ± 0.06a	0.21 ± 0.04b	0.36 ± 0.02a	0.32 ± 0.05b
13	2.83 ± 0.01a	1.16 ± 0.07b	2.40 ± 0.09a	0.80 ± 0.12b	0.44 ± 0.00a	0.39 ± 0.01b	0.34 ± 0.05a	0.29 ± 0.00b
14	3.25 ± 0.08a	1.26 ± 0.05b	1.56 ± 0.01a	0.85 ± 0.03b	1.73 ± 0.04a	0.35 ± 0.45b	0.35 ± 0.02a	0.32 ± 0.02b
15	3.25 ± 0.02a	1.35 ± 0.04b	2.74 ± 0.17a	1.38 ± 0.09b	0.50 ± 0.78a	0.86 ± 0.50b	0.31 ± 0.01a	0.23 ± 0.09b
16	3.46 ± 0.09a	1.65 ± 0.03b	2.96 ± 0.19a	1.51 ± 0.04b	0.52 ± 0.34a	0.16 ± 0.20b	0.32 ± 0.04a	0.27 ± 0.00b
17	2.70 ± 0.04a	1.33 ± 0.88b	2.54 ± 0.18a	1.29 ± 0.06b	0.16 ± 0.01a	0.06 ± 0.01b	0.30 ± 0.01a	0.24 ± 0.04b
18	3.46 ± 0.05a	1.68 ± 0.09b	2.37 ± 0.11a	1.58 ± 0.02b	1.08 ± 0.02a	0.09 ± 0.01b	0.36 ± 0.00a	0.32 ± 0.01b
19	3.02 ± 0.01a	1.29 ± 0.05b	2.67 ± 0.24a	1.18 ± 0.03b	0.37 ± 0.01a	0.16 ± 0.01b	0.24 ± 0.01a	0.11 ± 0.09b
20	3.25 ± 0.09a	1.80 ± 0.09b	2.88 ± 0.32a	1.65 ± 0.03b	0.36 ± 0.09a	0.12 ± 0.08b	0.36 ± 0.03a	0.29 ± 0.05b
21	3.30 ± 0.00a	1.48 ± 0.09b	2.76 ± 0.10a	1.26 ± 0.04b	0.54 ± 0.09a	0.20 ± 0.01b	0.37 ± 0.01a	0.30 ± 0.02b
22	3.46 ± 0.03a	1.67 ± 0.02b	2.39 ± 0.01a	1.58 ± 0.05b	1.07 ± 0.04a	0.09 ± 0.00b	0.35 ±0.06a	0.35 ± 0.00b
23	3.03 ± 0.02a	1.39 ± 0.01b	2.68 ± 0.04a	1.15 ± 0.01b	0.39 ± 0.02a	0.18 ± 0.05b	0.24 ± 0.01a	0.12 ± 0.04b
24	3.27 ± 0.00a	1.85 ± 0.09b	2.92 ± 0.05a	1.70 ± 0.02b	0.37 ± 0.04a	0.11 ± 0.02b	0.36 ± 0.01a	0.28 ± 0.01b

Tabla S2. Continuation of characterization of culture media used in the optimization of β-glucans from *Rhizopus oryzae* M10A1.

Run	Nitrogen (g/100mL)		Sugar total (mg/100mL)		Reducing sugars (mg/100mL)		Starch total (mg/100mL)	
	Before cultivation	After cultivation	Before cultivation	After cultivation	Before cultivation	After cultivation	Before cultivation	After cultivation
1	0.35 ± 0.00a	0.04 ± 0.00b	177.16 ± 0.35a	6.58 ± 0.24b	54.65 ± 0.72a	15.67 ± 0.70b	4342.11 ± 0.61a	461.15 ± 0.58b
2	0.47 ± 0.01a	0.21 ± 0.01b	166.25 ± 0.01a	13.22 ± 0.11b	52.18 ± 0.60a	13.02 ± 0.48b	3931.08 ± 0.30a	530.08 ± 0.53b
3	0.34 ± 0.01a	0.26 ± 0.01b	340.03 ± 0.05a	5.75 ± 0.47b	67.29 ± 0.36a	12.93 ± 0.81b	3621.55 ± 0.30a	436.09 ± 0.58b
4	0.44 ± 0.00a	0.24 ± 0.00b	351.97 ± 0.95a	10.07 ± 0.10b	63.28 ± 0.35a	18.13 ± 0.14b	3615.29 ± 0.17a	517.54 ± 0.72b
5	0.33 ± 0.00a	0.30 ± 0.00b	115.38 ± 0.07a	38.82 ± 0.99b	58.92 ± 0.72a	10.87 ± 0.30b	3418.13 ± 0.19a	663.74 ± 0.47b
6	0.32 ± 0.00a	0.30 ± 0.01b	389.88 ± 0.40a	57.68 ± 0.55b	44.76 ± 0.36a	7.71 ± 0.12b	3940.27 ± 0.35a	755.64 ± 0.00b
7	0.29 ± 0.00a	0.22 ± 0.00b	226.73 ± 0.40a	67.39 ± 0.23b	43.91 ± 0.73a	4.61 ± 0.36b	4024.10 ± 0.30a	411.03 ± 0.86b
8	0.31 ± 0.00a	0.28 ± 0.01b	236.42 ± 0.70a	90.12 ± 0.54b	43.11 ± 0.60a	9.73 ± 0.14b	3107.52 ± 0.71a	659.57 ± 0.09b
9	0.37 ± 0.01a	0.29 ± 0.01b	233.66 ± 0.35a	6.25 ± 0.71b	55.11 ± 0.97a	10.12 ± 0.05b	3940.31 ± 0.31a	400.58 ± 0.23b
10	0.36 ± 0.05a	0.27 ± 0.00b	232.01 ± 0.55a	6.20 ± 0.10b	54.12 ± 0.99a	10.01 ± 0.09b	3243.10 ± 0.09a	410.34 ± 0.45b
11	0.35 ± 0.05a	0.26 ± 0.02b	234.87 ± 0.15a	6.30 ± 0.05b	55.01 ± 0.35a	10.13 ± 0.10b	3145.09 ± 0.42a	435.98 ± 0.01b
12	0.36 ± 0.04a	0.28 ± 0.04b	231.10 ± 0.66a	6.24 ± 0.03b	54.09 ± 0.45a	10.09 ± 0.05b	3228.74 ± 0.26a	401.45 ± 0.00b
13	0.34 ± 0.09a	0.03 ± 0.00b	175.09 ± 0.25a	6.55 ± 0.04b	55.01 ± 0.00a	14.47 ± 0.17b	4339.11 ± 0.11a	401.50 ± 0.68b
14	0.45 ± 0.05a	0.20 ± 0.01b	162.25 ± 0.18a	12.99 ± 0.31b	51.69 ± 0.20a	12.59 ± 0.38b	3939.20 ± 0.99a	519.98 ± 0.55b
15	0.32 ± 0.09a	0.27 ± 0.01b	339.99 ± 0.50a	5.85 ± 0.09b	66.99 ± 0.16a	11.98 ± 0.01b	3611.05 ± 0.31a	433.00 ± 0.18b
16	0.42 ± 0.03a	0.22 ± 0.00b	350.99 ± 0.65a	10.00 ± 0.51b	64.00 ± 0.35a	18.03 ± 0.18b	3615.19 ± 0.17a	516.99 ± 0.72b
17	0.31 ± 0.00a	0.29 ± 0.09b	116.08 ± 0.15a	37.99 ± 0.02b	58.12 ± 1.12a	10.79 ± 0.10b	3399.99 ± 0.66a	660.67 ± 0.67b
18	0.31 ± 0.09a	0.29 ± 0.02b	390.01 ± 0.55a	56.98 ± 0.75b	45.00 ± 0.19a	7.70 ± 0.19b	3941.01 ± 0.15a	756.04 ± 0.10b
19	0.28 ± 0.03a	0.23 ± 0.05b	225.91 ± 0.63a	68.01 ± 0.13b	44.00 ± 0.22a	4.60 ± 0.16b	4023.10 ± 0.09a	412.02 ± 0.99b
20	0.30 ± 0.09a	0.27 ± 0.01b	237.12 ± 0.89a	90.02 ± 0.10b	43.01 ± 0.30a	8.99 ± 0.19b	3108.02 ± 0.01a	650.97 ± 0.01b
21	0.35 ± 0.09a	0.27 ± 0.03b	232.01 ± 0.25a	6.22 ± 0.00b	54.01 ± 0.55a	10.04 ± 0.04b	3335.61 ± 0.45a	415.78 ± 0.08b
22	0.36 ± 0.05a	0.28 ± 0.01b	234.00 ± 0.19a	6.26 ± 0.10b	54.99 ± 0.01a	10.16 ± 0.04b	3939.99 ± 0.21a	401.02 ± 0.03b
23	0.34 ± 0.07a	0.25 ± 0.01b	235.01 ± 0.05a	6.29 ± 0.15b	54.96 ± 0.65a	10.19 ± 0.25b	3146.99 ± 0.12a	435.49 ± 0.09b
24	0.36 ± 0.06a	0.29 ± 0.09b	231.10 ± 0.66a	6.25 ± 0.67b	54.09 ± 0.15a	10.09 ± 0.23b	3228.74 ± 0.16a	401.45 ± 0.00b

The values correspond to the average of three determinations ± the standard deviation. Different letters in the same row indicate significant differences at a confidence level of 95%.
